# Stepwise super minimally invasive full-thickness resection of early rectal cancer: based on the handmade rubber traction technique

**DOI:** 10.1055/a-2743-2258

**Published:** 2025-12-03

**Authors:** Qun Shao, Yaoqian Yuan, Shuai Tian, Kunming Lv, Enqiang Linghu, Qianqian Chen

**Affiliations:** 1 104607 Department of Gastroenterology, The First Medical Center of Chinese PLA General Hospital, Beijing, China; 2 Department of Gastroenterology, 970 Hospital of the PLA Joint Logistic Support Force, Yantai, China

Transanal endoscopic resection of early rectal cancer, particularly T1b lesions, has garnered increasing attention due to the challenges of deep-layer dissection 1 2 . Commonly used techniques include endoscopic full-thickness resection (EFTR) and endoscopic intermuscular dissection 1 2 . However, the precise identification of the fourth to fifth layers of the digestive tract wall and secure closure of large full-thickness defects remain significant technical hurdles. In our prior research, we implemented a handmade rubber loop-tissue clip traction method to facilitate wound approximation, yielding favorable outcomes 3 . In this case report, we applied this traction device to assist in digestive wall layer identification and to enable full-thickness defect closure via triangular configuration-mediated size reduction.

However, secure closure of large full-thickness defects – especially within the anatomical constraints of the low rectum – remains a significant technical challenge. Existing closure devices (e.g., over-the-scope clips and clip-loop systems) are limited by cost, accessibility, and technical complexity. Herein, we describe a stepwise full thickness super minimally invasive resection (sft-SMIR) technique for early rectal cancer, featuring a handmade rubber loop that serves a pivotal dual function, enabling precise full-thickness resection via traction-mediated dissection and facilitating defect closure through a novel triangular traction configuration. We used transanal sft-SMIR surgery to remove the lesion ( Fig. 1 and Video 1 ).

**Fig. 1:**
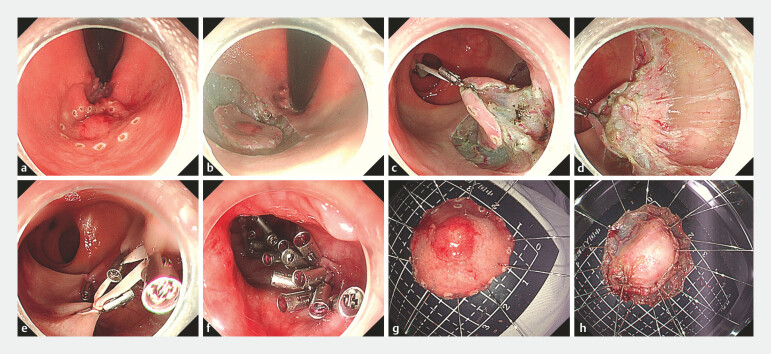
sft-SMIR with the handmade rubber traction technique for early rectal cancer. **a** Circumferential electrocautery marking around the rectal lesion. **b** Circumferential mucosal incision using an electrosurgical knife, followed by submucosal dissection. **c** Placement of clips and the handmade rubber loop to tract the lesion edge to expose the muscularis propria. **d** Stepwise full-thickness and En bloc resection of the lesion using a triangular knife, confirming full-thickness excision (visible adipose tissue). **e** Application of tissue clips and the rubber loop to form triangular traction, reducing the defect size. **f** Complete full-thickness closure of the defect using clips, with removal of the rubber loop after secure closure. **g** The mucosal layer of the postoperative gross specimen (size 3.0 × 3.0 cm). **h** The serosal layer of the postoperative gross specimen (EFTR size 2.2 × 2.0 cm). sft-SMIR, stepwise full thickness super minimally invasive resection.

sft-SMIR with the handmade rubber traction technique for early rectal cancer. sft-SMIR, stepwise full thickness super minimally invasive resection.Video 1

A 60-year-old woman was admitted to the hospital due to a T1-stage rectal space-occupying lesion located 3 cm from the anus. Then, she underwent transanal sft-SMIR surgery. The procedure entailed circumferential electrocautery marking and submucosal injection to lift the lesion, followed by mucosal incision and submucosal dissection to approach the target area ( Fig. 1 **a, b** ). Utilizing tissue clips and a rubber loop for triangular traction to optimize exposure, a triangular knife was employed to sequentially incise the circular muscle layer, longitudinal muscle layer, and serosal layer, ultimately achieving en bloc full-thickness resection of the lesion with meticulous hemostasis ( Fig. 1 **c, d** ). The postoperative defect was approximately 4.0 × 3.5 cm. We used a rubber loop traction technique to form a triangle to shorten the wound defect ( Fig. 1 **e** ). When most of the partially resected mucosal layers covered the defect, we continued to use traditional clips to close the defect along both sides ( Fig. 1 **f** ). Subsequently, the handmade rubber loop was removed. The specimen is fixed and photographed with both sides ( Fig. 1 **g, h** ). The patient received postoperative treatment including fasting, acid suppression, gastric protection, fluid replacement, infection prevention (6 d of ornidazole plus cefmetazole sodium), and nutritional support. The patient recovered well with no adverse events such as bleeding or fever, and was discharged from the hospital on the seventh day after surgery. Two months later, an in-hospital colonoscopy showed that the postoperative wound had healed well.

The integration of the sft-SMIR technique with a handmade rubber traction device represents a multifaceted clinical innovation. Its core advantage resides in the dual functionality of the handmade rubber loop: (1) During resection, the rubber loop – anchored via tissue clips – facilitates multidirectional traction, significantly enhancing visualization of the low rectal surgical field. This enables precise layered dissection of the rectal wall (from the circular muscle layer through the longitudinal muscle layer to the serosal layer), ensuring anatomical precision for radical resection of T1-stage tumors. (2) During defect closure, the triangular traction system – constructed using three tissue clips – actively reduces the full-thickness defect area, transforming complex defects into a linear, approximable configuration amenable to reliable closure with conventional tissue clips.

Endoscopy_UCTN_Code_TTT_1AQ_2AD_3AD

## Notes

Endoscopy E-Videos https://eref.thieme.de/e-videos
*E-Videos* is an open access online section of the journal *Endoscopy* , reporting on interesting cases and new techniques in gastroenterological endoscopy. All papers include a high-quality video and are published with a Creative Commons CC-BY license. Endoscopy E-Videos qualify for HINARI discounts and waivers and eligibility is automatically checked during the submission process. We grant 100% waivers to articles whose corresponding authors are based in Group A countries and 50% waivers to those who are based in Group B countries as classified by Research4Life (see: https://www.research4life.org/access/eligibility/ ). This section has its own submission website at https://mc.manuscriptcentral.com/e-videos .

## References

[1] Ichimasa K Kudo SE Tan KK Challenges in Implementing Endoscopic Resection for T2 Colorectal Cancer Gut Liver 2023 18 218 10.5009/gnl230125 37842729 PMC10938148

[2] Ichimasa K Kudo SE Hayashi T Potential indications for peranal endoscopic myectomy in lower rectal cancer Gastrointest Endosc 2025 101 1244 1247 10.1016/j.gie.2025.02.012 39956466

[3] Yuan Y Lv K Ning B Application of handmade rubber loop traction assisted defect closure after super minimally invasive surgery of gastric gastrointestinal stromal tumor Endoscopy 2025 57 E421 E422 10.1055/a-2589-1585 40389244 PMC12088867

